# Sequence capture by hybridization reveals elusive hydrocarbon degradation potential

**DOI:** 10.1111/1751-7915.12591

**Published:** 2017-01-20

**Authors:** Frederick von Netzer

**Affiliations:** ^1^ Department of Civil and Environmental EngineeringUniversity of WashingtonSeattleWAUSA

## Abstract

Highlight description of article (Ranchou‐Peyrouse et al.) to be published in MBT.

The paper of Ranchou‐Peyruse *et al*. (2016) presents evidence of hydrocarbon degraders present in formation water from a depth of 853 m in the Paris Basin in France. Such deep formation waters are used for storing natural gas. This is a challenging environment for sampling, considering that at the designated depths, the acquisition of sediments is difficult and often beyond the funding of most research projects. Therefore, the scientists were forced to utilize the biomass available in pumped groundwater. In this particular case, 500 l of groundwater had to be collected and filtered to obtain enough biomass for the characterization of the microbial community.

The authors tried to characterize the microbial community through biodegradation assays as well as established molecular methods for phylogenetic and functional gene markers. A part of the biomass gained through filtration was incubated anaerobically with toluene and xylene isomers as model compounds commonly found in natural gas storage. For the molecular characterization, the authors used 16S as the phylogenetic and fumarate‐adding enzyme genes as the functional gene marker for hydrocarbon degradation.

The group of fumarate‐adding enzymes contains the benzylsuccinate synthases, the alkyl‐ and methylnaphthylsuccinate synthases. These enzymes activate methylated aromatic, methylated polyaromatic and aliphatic hydrocarbons under anaerobic conditions. For the genes of the different enzymes, many primer sets were published over the last years (von Netzer *et al*., 2016), contributing to unravelling novel lineages of fumarate‐adding enzymes potentially involved in anaerobic hydrocarbon degradation.

However, the different hydrocarbon fumarate‐adding enzymes are a highly diverse and polyphyletic group, spanning across different phylogenetic classes, compounds and respiration modes. Therefore, it is possible to still get negative results even with the multitude of established primers and probes. This is due to PCR artefacts which are difficult to avoid while attempting to amplify low‐abundance functional genes and targets which are more remote from the classical benzyl succinate synthase lineages.

The incubation experiment of Ranchou‐Peyruse *et al*. (2016), patiently performed with biomass gained from filtrated formation water over several years, showed reproducible degradation of toluene and *o*,*m*‐xylene under sulphate‐reducing conditions, but the authors were not able to detect the involved functional genes with the aforementioned PCR methods. The authors then turned to a sequence capture hybridization method (established by Denonfoux *et al*. (2013) for detecting the gene for the methyl coenzyme M reductase subunit A) to detect the otherwise evasive benzylsuccinate synthase genes. They designed oligomers targeting benzylsuccinate synthase genes with PCR tags. The targeted gene fragments from the extracted genetic material hybridizes to these probes and can thus be amplified. This hybridization–amplification step can be repeated with the captured products to increase the sequence yield before submitting to next‐generation sequencing for final sequence identification.

In this manner, Ranchou‐Peyruse *et al*. (2016) managed to assign the genes involved in toluene and xylene degradation to clostridial benzylsuccinate synthase genes and related homologues. The 16S characterization showed that the community found in the formation water was dominated by *Firmicutes* belonging to the families of *Thermoanaerobacterace* and *Peptococcaceae*. In particular, for the latter family, there is an increased number of recent reports of their importance for subsurface ecology and anaerobic hydrocarbon degradation as summarized by Lueders (2017).

This research paper shows that the application of the sequence capture hybridization can be more sensitive than the usual molecular methods for detecting rare sequences. In this case, the incubation experiment hinted towards the presence of anaerobic toluene and xylene degrades, but established primer sets were not up to the task to amplify the involved functional genes.
